# Angelica Polysaccharide Antagonizes 5-FU-Induced Oxidative Stress Injury to Reduce Apoptosis in the Liver Through Nrf2 Pathway

**DOI:** 10.3389/fonc.2021.720620

**Published:** 2021-08-16

**Authors:** Di Zeng, Yaping Wang, Yi Chen, Danyang Li, Guoli Li, Hanxianzhi Xiao, Jiyin Hou, Ziling Wang, Ling Hu, Lu Wang, Jing Li

**Affiliations:** ^1^Laboratory of Stem Cells and Tissue Engineering, Department of Histology and Embryology, Chongqing Medical University, Chongqing, China; ^2^Centre for Lipid Research & Key Laboratory of Molecular Biology for Infectious Diseases, Chongqing Medical University, Chongqing, China

**Keywords:** 5-fluorouracil, ASP, oxidative stress, Nrf2, hepatotoxicity, apoptosis

## Abstract

Oxidative stress induced by chemotherapeutic agents causes hepatotoxicity. 5-Fluorouracil (5-FU) has been found to have a variety of side effects, but its toxic effect on the liver and the mechanism are still unclear. Angelica polysaccharide (ASP), the main active ingredient of Dang Gui, has antioxidative stress effects. In this study, we investigated the antagonistic effects of ASP on 5-FU-induced injury in the mouse liver and human normal liver cell line MIHA and the possible mechanism. Our results show that ASP inhibited 5-FU-induced the decrease in Bcl-2 protein and the increase in Bax protein. ASP alleviated 5-FU-induced the increase in alanine aminotransferase (ALT), triglyceride (TG), and aspartate aminotransferase (AST) content; hepatic steatosis; and liver fibrosis. ASP restored 5-FU-induced swelling of mitochondria and the endoplasmic reticulum. 5-FU promoted the expression of Keap1 and increased the binding to NF-E2-related factor 2 (Nrf2) to reduce the nuclear translocation of Nrf2, thereby weakening the transcriptional activity of Nrf2 to inhibit the expression of HO-1; reducing the activity of GSH, SOD, and CAT to increase ROS content; and aggravating DNA damage (indicated by the increase in 8-OHdG). However, ASP reversed these reactions. In conclusion, ASP attenuated the 5-FU-induced Nrf2 pathway barrier to reduce oxidative stress injury and thereby inhibit the disorder of lipid anabolism and apoptosis. The study provides a new protectant for reducing the hepatic toxicity caused by 5-FU and a novel target for treating the liver injury.

**Graphical Abstract:**
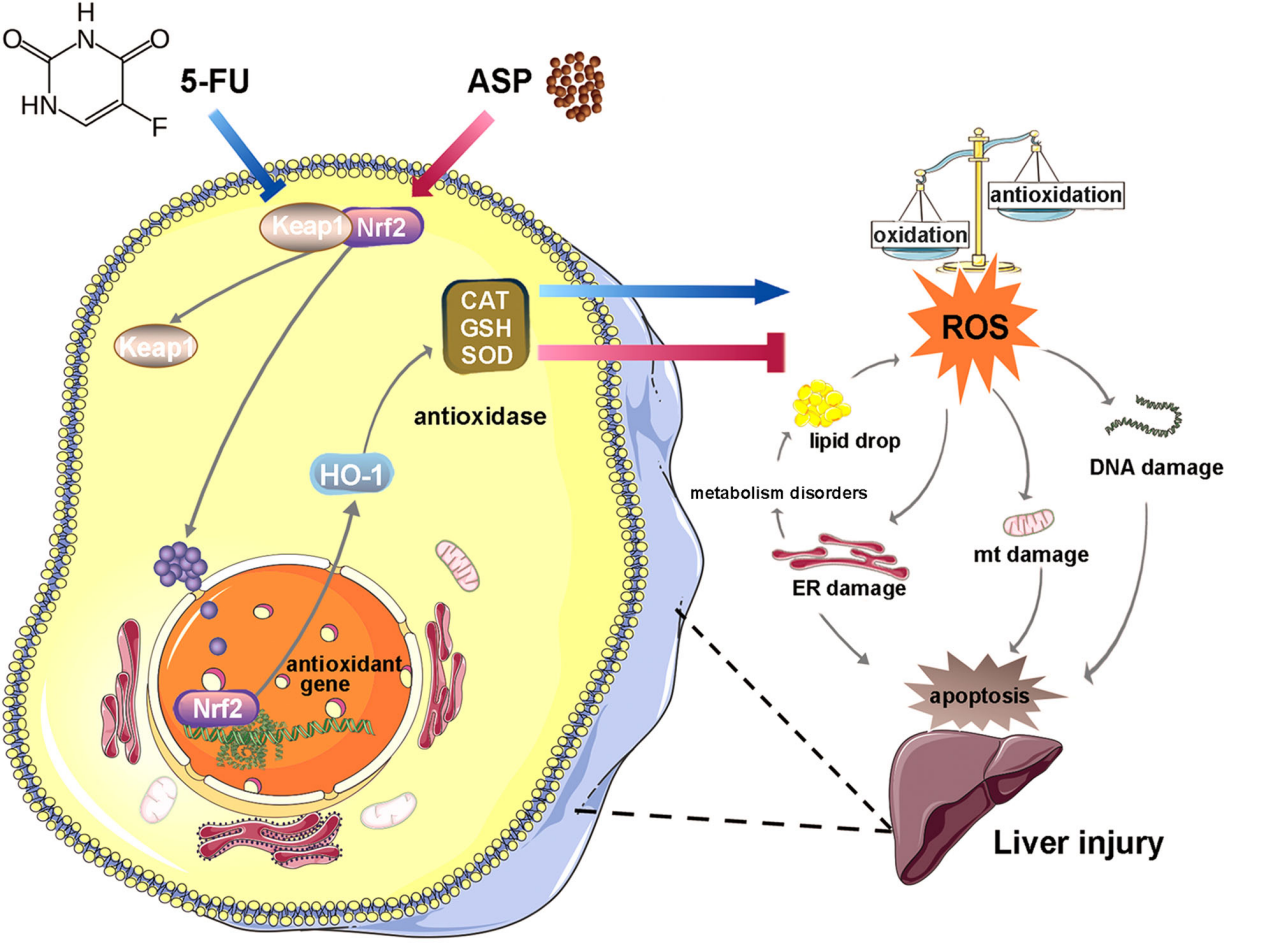


## Introduction

5-Fluorouracil (5-FU) is a cytotoxic chemotherapy medication used to treat cancer, such as colorectal cancer, oesophageal cancer and stomach cancer, with the effect of blocking thymidylate synthase (1, 2). Administration of 5-FU results in a deficiency of deoxythymidine monophosphate, so that rapidly dividing cancer cells undergo cell death by thymineless death (3). The typical adverse effects of 5-FU are nausea, vomiting, mucositis, and diarrhoea (4). In addition, 5-FU can promote the production of free radicals and cause mitochondrial damage, leading to cardiotoxicity (5). Also, oxidative stress is vital in mediating 5-FU-induced renal injury (6). However, there are very few reports on the liver toxicity caused by 5-FU. A study has shown that after intraperitoneal injection of 5-FU, there are pathological and ultrastructural abnormalities of liver tissue (7), but the possible mechanism has not been elucidated.

Factors such as autoimmune attacks of liver cells, viral infections and drug abuse can lead to liver injury (8). Liver injury is regulated by multiple factors, and the mechanism of its pathogenesis and development has not been fully elucidated. Reactive oxygen species (ROS) are potentially vital in cell signaling and disease. The excessive deposition of free fatty acids and triglycerides (TGs) will lead to liver lipid metabolism disorders, which increase the liver’s susceptibility to oxidative stress (OS), resulting in a large amount of ROS, which leads to apoptosis (9). Additionally, some antioxidants have been shown to improve lipotoxicity (10). A study found that inhibiting the inflammatory apoptotic response and the production of ROS could improve liver apoptosis in mice (11, 12). In addition, inhibiting the production of liver ROS could ameliorate liver fibrosis (13). Therefore, reducing the production of ROS to reduce liver injury is very important.

Various biological processes of the liver involve the Nrf2 antioxidant system, such as regulating the metabolism of liver substances, detoxification, and regeneration of liver cells, which are potential targets for treating liver diseases and liver injury (14). Nrf2 is a crucial transcription factor with an antioxidant effect (15). Under normal conditions, keap1 and Nrf2 exist as dimers in the cytoplasm. Once exposed to oxidative stress, the two dissociate and Nrf2 is transcribed to regulate the transcriptional activation of a series of cytoprotective genes (16). HO-1 is one of the antioxidant response kinases downstream of Nrf2. It has the ability to reduce liver damage (17). Studies suggest that the activation of Nrf2 reduces liver injury (18–20). There has been an increase in interest in finding potential protective agents against adverse reactions associated with chemotherapy. Adjuvant therapy with drugs with antioxidant function and minor side effects may be an effective measure to reduce the hepatotoxicity caused by 5-FU.

Angelica polysaccharide (ASP) is the main active component in Dang Gui with the functions of antioxidation, immune regulation, and radiation protection (21–24). Our previous study showed that ASP slows down hematopoietic stem cells senescence by reducing oxidative stress (25); our other study showed that ASP could also antagonize the inhibitory effect of 5-FU on bone marrow stromal cells (2). The damage to some organs induced by 5-FU is closely related to oxidative stress, so we hypothesize that 5-FU could aggravate oxidative stress and lead to liver injury, while ASP could activate the antioxidant response, promote ROS clearance, and reduce lipid deposition and cell apoptosis to prevent 5-FU-induced liver injury.

## Materials and Methods

### Reagents and Antibodies

5-FU (F6627-1G, purity ≥ 99%) was purchased from Sigma-Aldrich (Saint Louis, Missouri, USA) and dissolved in dimethyl sulfoxide and phosphate-buffered saline (PBS). Angelica polysaccharide was purchased from Shanxi Ciyuan Biotechnology Co. Ltd. (Xi’an, China) with a purity of ≥ 95%, dissolved in normal saline (NS). The primary antibody used are as follows: HO-1 (Abcam, cat. no. ab68477, Cambridge, UK), Nrf2 (CST, cat. no. D1Z9C, Danvers, MA, USA), Keap1 (CST, cat. no. D6B12), β-actin (CST, cat. no.13E5), Bcl-2 (Santa Cruz, cat. no.7382, CA, USA), Bax (Santa Cruz, cat. no.20067), 8-OHdG (Bioss, cat. no.1278R, Beijing, China), and Lamin B1 (Proteintech, cat. no. 66095, PA, USA).

### Animals and Treatments

C57BL/6J mice (male, 6-8 weeks old) were obtained from the Laboratory Animal Center of Chongqing Medical University (*n* = 60). The mice were randomly divided into four groups: the control group (*n* = 15), ASP group (*n* = 15), 5-FU group (*n* = 15), and 5-FU + ASP group (*n* = 15). In the 5-FU group, 5-FU (150 mg/kg) was injected intraperitoneally into mice, and after 6 h, normal saline (10 mL/kg/d) was injected for 7 d. In the 5-FU + ASP group, after 6 h of 5-FU injection, ASP (100 mg/kg/day) was injected intraperitoneally daily for 7 d. In the ASP group, ASP (100 mg/kg/day) was injected for 7 d. All control animals were given 10 mL/kg/d normal saline intraperitoneally for 7 d. All animal experiments were approved by the Chongqing Medical University Animal Care and Use Committee and performed following institutional and national guidelines.

### Cell Culture and Culture Condition

The human normal hepatic MIHA cell line was a gift from the Centre for Lipid Research & Key Laboratory of Molecular Biology for Infectious Diseases (Chongqing, China). Cells were cultured in DMEM medium supplemented with 10% fetal bovine serum and 100 μg/mL penicillin and streptomycin and incubated at 37°C in 5% CO_2_ as described (26).

### Biochemical Analysis

After the mice were sacrificed, an appropriate amount of tissue samples was taken, added to precooled PBS, homogenized in an ice bath, and centrifuged for 10 min at 4°C. The BCA kit (Beyotime Institute of Biotechnology, Shanghai, China) was used to detect the protein concentration in the supernatant. After that, the activity of glutathione (GSH), catalase (CAT), and superoxide dismutase (SOD), and the contents of alanine aminotransferase (ALT), aspartate aminotransferase (AST), total cholesterol (TC), triglyceride (TG), malondialdehyde (MDA), and nitric oxide (NO) were determined with the corresponding biochemical kits (Jian Cheng Biotechnology, Nanjing, China) as previously described by our laboratory (27). All procedures were performed based on the manufacturer’s protocols (28).

### Measurement of Reactive Oxygen Species

ROS content was detected by a DCFH-DA probe (Sigma-Aldrich, MO, USA). After incubation with ROS staining solution for 30 min at 37°C, MIHA cells or liver sections were washed three times with PBS. The fluorescence was detected by a fluorescence microplate reader for cells or microscope for liver sections (Olympus, Tokyo, Japan).

### Ultrastructure of the Mice Liver

1mm^3^ of the fresh liver was immediately immobilized in sufficient glutaraldehyde. Then dehydrated with graded ethanol and embedded within the epoxy resin. After polymerization, 70 nm ultrathin sections were cut and then stained with uranyl acetate and lead citrate as described (28). The cells were observed by transmission electron microscopy (TEM).

### Histopathological Staining

Paraffin sections of liver tissue were prepared with a thickness of 5 μm. Tissue sections were stained with hematoxylin-eosin (HE) to assess the general liver structure as previously described by our laboratory (28). Cells were also stained with a Masson kit (Solabio, Beijing, China) to measure changes in collagen fibers. Images were obtained using microscopy (Olympus, Tokyo, Japan).

### TUNEL Assay

Paraffin sections of liver tissue were prepared, and the InSitu Cell DeathDetection Kit (Roche) was used to conduct experiments according to the instructions. The nuclei of apoptotic cells were dark brown. The sections were stained lightly with hematoxylin as described (29). Images were obtained using microscopy (Olympus, Tokyo, Japan).

### Oil Red O Staining

The frozen liver sections of 8μm thick, and cells were stained with Oil Red O (Sigma, USA) for 10 min, washed off the dye solution, and stained with hematoxylin for 1 min. The slides were fixed with glycerol and phosphate buffered saline (glycerol: PBS = 1:1) and observed under a microscope (Olympus, Tokyo, Japan).

### Immunohistochemistry Staining

The immunohistochemistry SP9000 kit (Zhongshan Chemical Industry, Beijing, China) was used for the experiment. The primary antibody against Keap1 was added and incubated at 4°C overnight. The sections were reheated at 37°C for 30 min. The secondary antibody was added and incubated for 30 min. Incubated with streptavidin-HRP at room temperature for 30 min and then incubated with diaminobenzidine stained substrate and stained with Mayer’s hematoxylin, as we did before (26). Keap1 proteins were represented as brown or yellow granular clumps. Images were obtained using microscopy (Olympus, Tokyo, Japan).

### Reverse Transcription-Quantitative PCR Assay

RT-qPCR assay was performed in a Real-Time PCR Detection System (Bio-Rad Laboratories, Inc.) according to the procedures provided by the reagent provider (Bio-Rad Laboratories, Inc.) as we described (26). The relative quantification values for each gene were calculated by the 2 ^−ΔΔCt^ method using β-actin as an internal reference. The primers used (Sangon Biotech Co., Ltd. Shanghai, China) are shown in **Supplementary Table 1**.

### Immunoblot Assay

The protease inhibitor (TargetMol, USA) and Reagents kit (Thermo Fisher, USA) were used to extract nuclear and cytosolic protein fractions. The proteins were performed to SDS-PAGE. Next, the separated proteins were transferred to the polyvinylidene difluoride (PVDF) membrane. After blocking the PVDF membranes with 5% nonfat milk for 1 h, the primary antibodies against Bcl-2, Bax, Nrf2, Keap1 and HO-1 were added and incubated at 4°C overnight. And added the corresponding secondary antibody to the membranes and incubated for 1 h at room temperature. The immunoblots were analyzed using the ChemiDoc Touch Imaging System (Bio-Rad Laboratories, Inc.) as we described (26). The Image Lab 5.2.1 (BIO-RAD, Hercules, CA, USA) was used to analyze the intensities of the protein bands.

### Immunofluorescence Staining

Frozen sections of liver tissue and cells were fixed with paraformaldehyde. And the cells were permeabilized with 1% goat serum albumin blocking solution containing 0.5% Triton X-100 for 30 minutes. Then, primary antibodies against 8-OHdG, Nrf2, and HO-1 were added and incubated at 4°C overnight. Next, the sections and MIHA cells were stained with the secondary antibody for 1 h at 37°C. DAPI was used to stain the nuclei for 5 minutes (26). Images were captured with fluorescence microscopy (Olympus, Tokyo, Japan).

### Statistical Analysis

The data in the text are presented as the mean ± SD (standard deviation). Statistical analysis was performed with GraphPad Prism 7.0 software (San Diego, CA, USA). One-way ANOVA was used to determine the differences when there were four groups. Student’s t-test was used to evaluate the difference between when there were two groups. *p* < 0.05 was considered as statistically significant.

## Results

### ASP Alleviated 5-FU-Induced Histopathology and Apoptosis in the Liver of Mice

**Figure 1:**
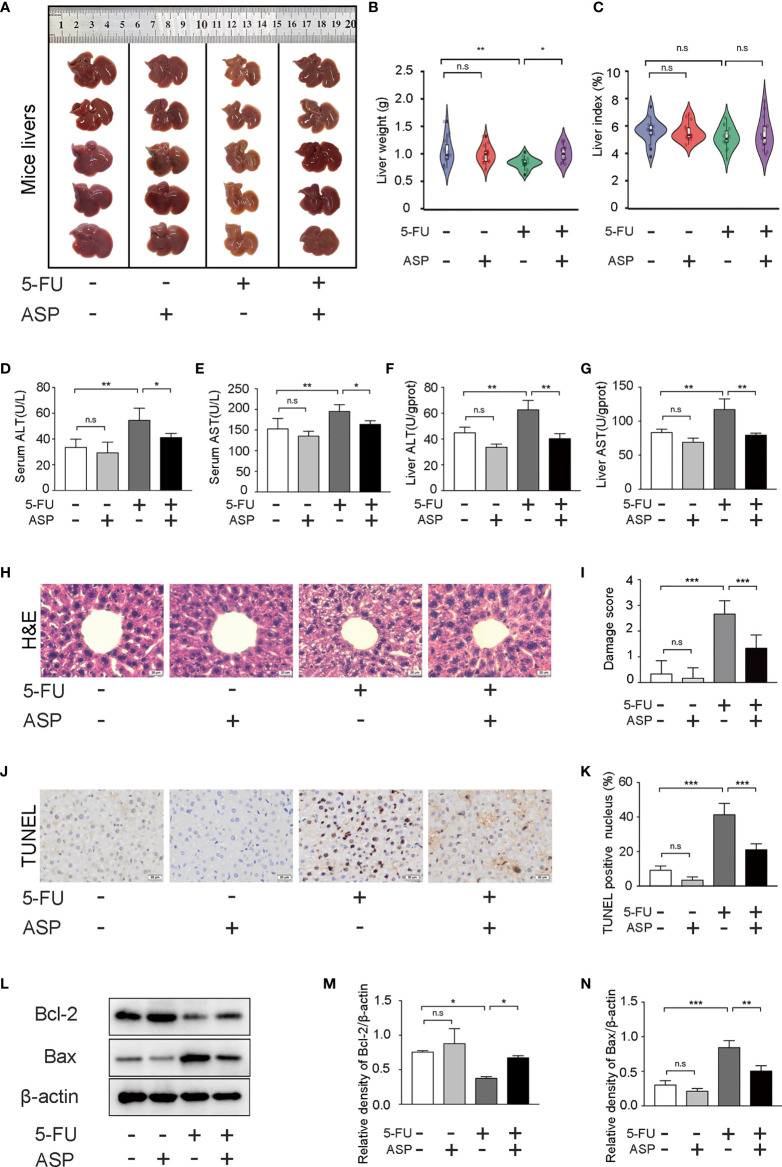
ASP attenuated 5-FU-induced liver histopathological damage and apoptosis in mice. **(A)** The appearance of the livers of each group was examined. **(B)** The weight of livers of each group was examined (*n* = 15/group). **(C)** The liver index was calculated by the ratio of the liver weight (g) to the mouse body weight (g) x 100 (*n* = 15/group). **(D, E)** The levels of ALT and AST in serum were detected (*n* = 5/group). **(F, G)** The levels of ALT and AST in mice liver were detected (*n* = 3/group). **(H, I)** Hematoxylin and eosin staining (magnification, × 400, scale bar, 20 μm). The stained sections were scored using a four-point scale from 0 to 3, with 0, 1, 2, and 3 representing no damage, mild damage, moderate damage, and severe damage, respectively (*n* = 6/group). **(J, K)** TUNEL staining and quantitative results of the percentage of TUNEL-positive cells in the liver of different experimental groups (magnification, × 400, scale bar, 20 μm). Images were analyzed using the image analysis program ImageJ (*n* = 6/group). **(L–N)** Western blot assay was performed to detect the levels of Bcl-2 and Bax. The intensity of the bands was calculated using ImageJ software. β-actin served as the internal control (*n* = 3/group). ^*^p < 0.05; ^**^p < 0.01; ^***^p < 0.001. n.s., not statistically significant.

The liver appearance of the mice was yellow-tinged and greasy in the 5-FU treatment group, while the livers of the other three groups were pink-red (**Figure 1A**). Additionally, the liver weight was decreased in the 5-FU group; however, ASP treatment restored the liver weight decrease induced by 5-FU (**Figure 1B**). Due to the weight loss of mice in the 5-FU group, even though the liver weight was reduced, the difference in the liver index between the groups was not statistically significant (**Figure 1C**). We then questioned whether treatment with ASP might alleviate liver damage. The results showed that ASP treatment alleviated the 5-FU-induced the increase of ALT and AST in the serum and liver (**Figures 1D–G**). Histological examination showed that hepatic lobules structure damage and vacuolization of hepatocytes in the 5-FU group (**Figures 1H, I**). Further analysis of the process of apoptosis showed that the count of TUNEL-positive cells in 5-FU-treated mice also decreased after ASP treatment (**Figures 1J, K**). The results indicated that the level of Bcl-2 decreased in the 5-FU treatment group, while the level of Bax was increased, and ASP treatment reversed these changes. 5-FU-induced hepatocyte apoptosis was inhibited after ASP treatment (**Figures 1L–N**). These results indicated that ASP effectively reduced the structural damage to liver tissue and liver apoptosis caused by 5-FU.

### ASP Attenuated 5-FU-Induced Liver Lipid Deposition *In Vivo*

**Figure 2:**
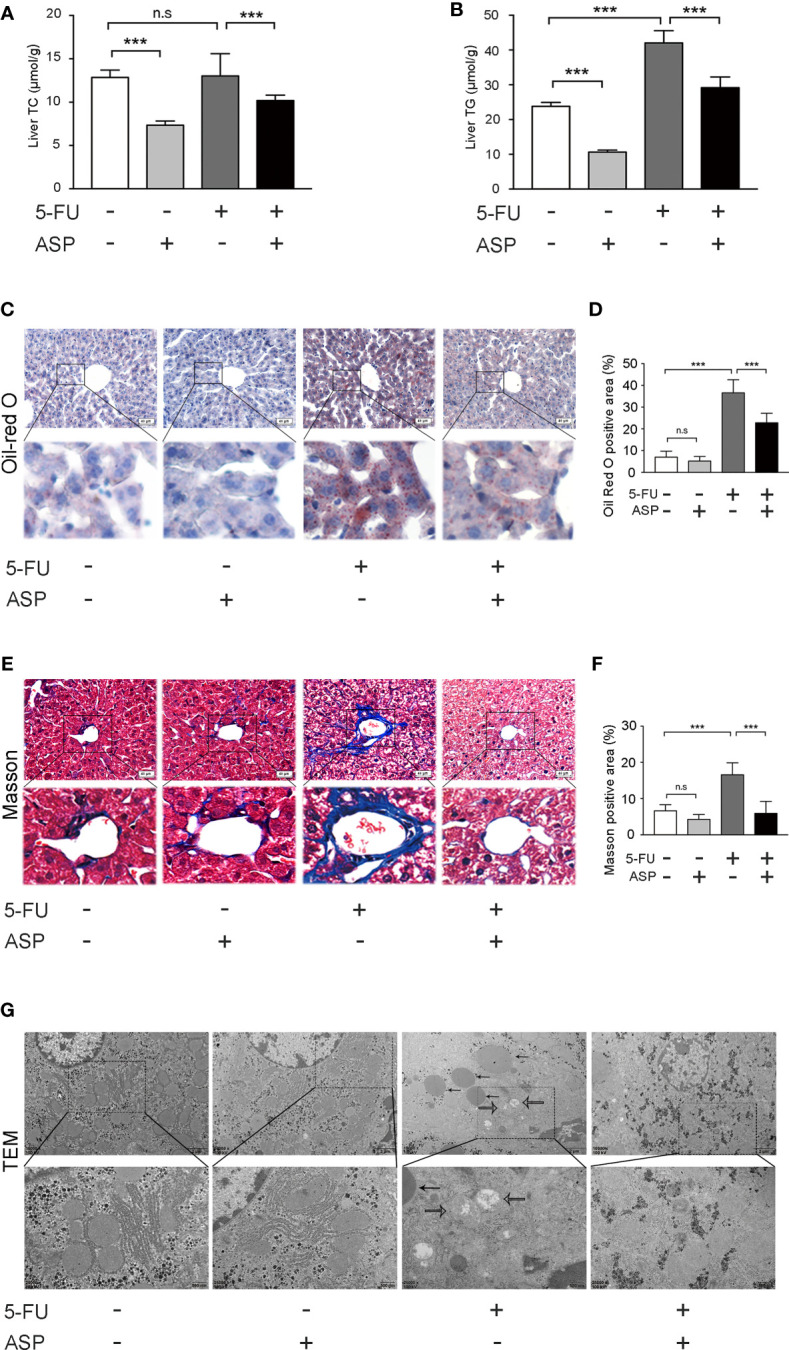
ASP attenuated 5-FU-induced liver lipid deposition *in vivo*. **(A)** The level of TC was measured. **(B)** ASP treatment reversed the 5-FU-induced abnormal elevation of liver biochemical parameters and TG (*n* = 6/group). **(C, D)** Oil Red O staining (nuclei were stained purple, and lipids were stained red). ASP rescued the lipogenesis induced by 5-FU treatment (magnification, × 200, scale bar, 40 μm). Images were analyzed using ImageJ (*n* = 6/group). **(E, F)** Masson staining of liver tissues (magnification, × 200, scale bar, 40 μm). Images were analyzed using ImageJ (*n* = 6/group) **(G)** TEM micrograph of the mice liver. The solid arrow points to the lipid droplet, and the open arrow points to the swollen mitochondria (magnification, × 10000 or × 25000, scale bar, 2 μm or 500 nm, respectively). ***p < 0.001. n.s., not statistically significant.

The biochemical indexes of liver tissue showed that the concentration of TG in mice liver in the 5-FU group was increased, and ASP treatment alleviated the abnormal increase in TG (**Figure 2B**). However, there was no significant difference in TC levels between the 5-FU group and the control group (**Figure 2A**). Oil Red O staining demonstrated more lipid deposition in the 5-FU group, whereas ASP treatment alleviated this abnormal phenomenon (**Figures 2C, D**). Masson staining showed the increasing area of fibrosis in the portal site of the mice liver in the 5-FU group, but ASP reduced this increased area of fibrosis (**Figures 2E, F**). Additionally, changes in liver ultrastructure were observed by electron microscopy, which showed that there was a large area of lipid deposition and mitochondrial swelling in the 5-FU group. Nevertheless, treatment with ASP alleviated lipid deposition and mitochondrial swelling (**Figure 2G**). These results suggested that ASP attenuates 5-FU-induced liver lipid deposition and mitochondrial swelling

### Effects of 5-FU Treatment on Lipid Synthesis, Lipid Uptake and Lipid-Metabolism-Related Gene Expression

**Figure 3:**
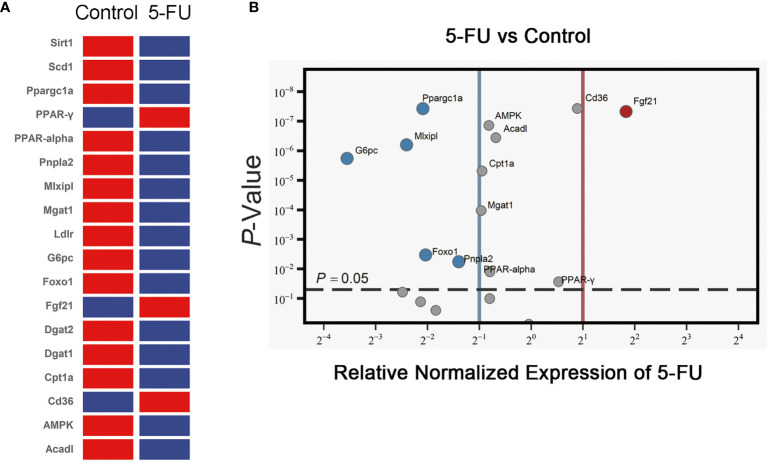
Effects of 5-FU treatment on lipid synthesis, lipid uptake, and lipid metabolism-related gene expression. **(A)** The expression of genes in the livers of mice was detected by RT-qPCR assay (*n* ≧ 6). Clustergram (Red means high mRNA expression level, and blue means low mRNA expression level). **(B)** Volcano ≧ plot (the left side of the solid blue line represents genes whose mRNA expression level has been reduced by more than two times, the right side of the solid red line represents genes whose mRNA expression level has increased more than twice, and the genes above the gray dotted line indicate that the difference is statistically significant, *p* < 0.05.

The expression of 18 genes related to lipid synthesis, uptake and metabolism was detected by RT-qPCR (**Figures 3A, B**). The expression of 3 genes in the 5-FU treatment group increased, and the expression of 15 genes decreased. The mRNA levels of Sirt1, Scd1, Dgat1, Dgat2, and Ldlr were not significantly different. The expression of PPAR-γ, CD36, and Fgf21 was increased significantly, while that of Ppargc1a, Mlxipl, G6pc, Foxo1, Pnpla2, AMPK, Acadl, Cpt1a, Mgat1, and PPAR-alpha was decreased. Among them, PPAR-alpha and Cpt1a are essential genes that regulate fatty acid β-oxidation. Ppargc1a is also a peroxisome proliferator-activated receptor coactivator. Pnpla2 is related to fatty acid hydrolysis. The results suggested that 5-FU-induced certain obstacles in the synthesis, uptake, and metabolism of lipids in the liver of mice, which may be related to the diminishment of fatty acid oxidation and hydrolysis in the liver.

### ASP Treatment Alleviated 5-FU-Induced Oxidative Damage in Mice

**Figure 4:**
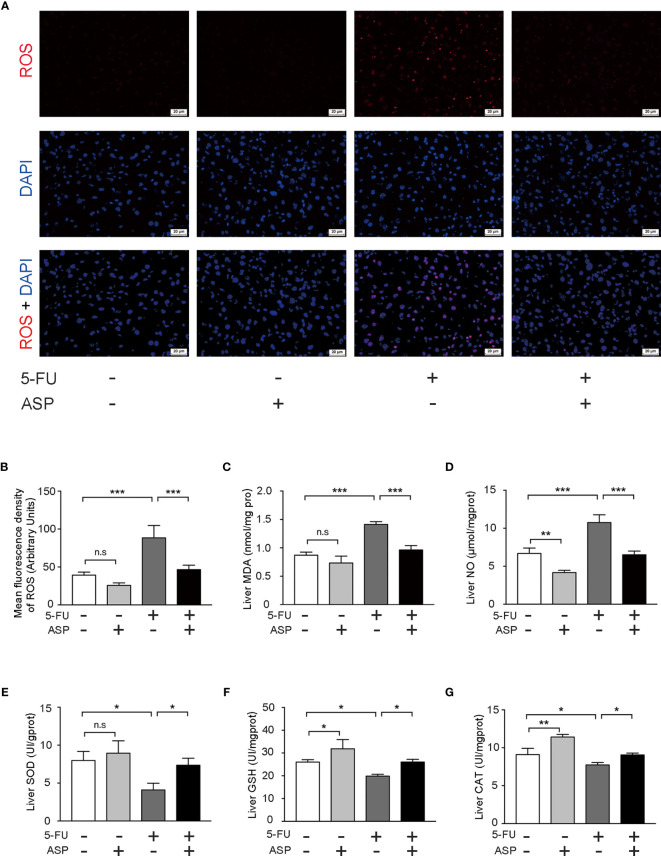
ASP treatment relieved 5-FU-induced oxidative injury in mice. **(A)** The level of ROS was determined (magnification, × 400, scale bar, 20 μm). **(B)** The intensity of fluorescence was quantified under microscopy and analyzed using the image analysis program ImageJ (*n* = 6/group). **(C, D)** ASP rescued the increased MDA and NO content caused by 5-FU in mice liver (*n* = 3/group). **(E–G)** ASP rescued the decreased levels of SOD, GSH, and CAT caused by 5-FU in mice liver (*n* = 3/group). ^*^p < 0.05; ^**^P < 0.01; ^***^p < 0.001. n.s., not statistically significant.

We further detected the oxidative and antioxidant balance in each group. The results demonstrated that ASP treatment alleviated the increase in ROS levels induced by 5-FU (**Figures 4A, B**). In addition, the activities of CAT, GSH and SOD were detected. Also, the contents of MDA and NO were detected. ASP rescued the increase in MDA and NO content caused by 5-FU (**Figures 4C, D**) and recovered the decreased levels of SOD, GSH and CAT caused by 5-FU in the livers of mice (**Figures 4E–G**). The results indicated that ASP treatment could reduce 5-FU-induced liver oxidative damage in mice.

### ASP Rescued the Inhibitory Effect of 5-FU on Nrf2 Pathway *In Vivo*

**Figure 5:**
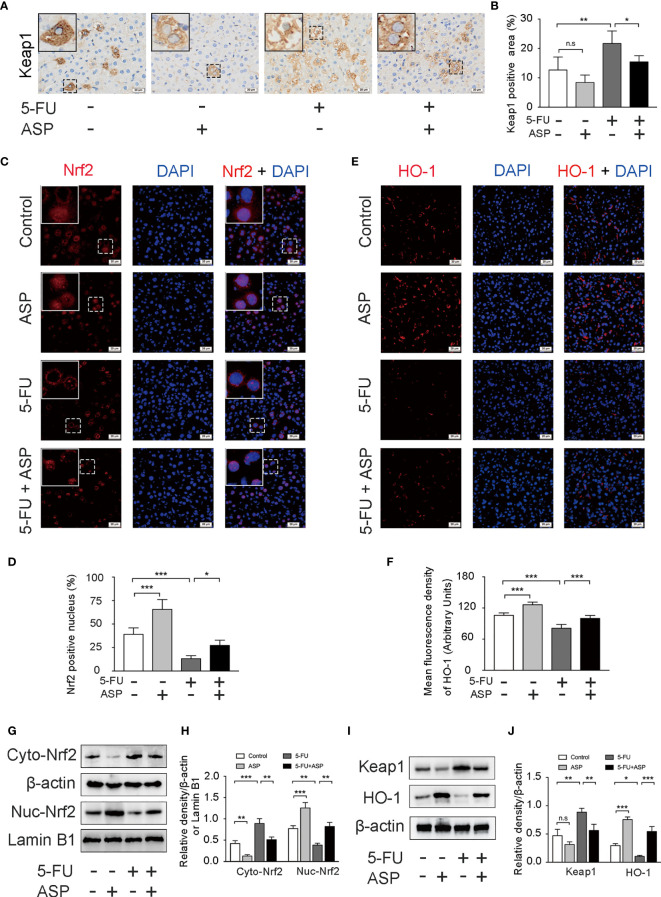
ASP rescued the inhibition of the Keap1- Nrf2 pathway caused by 5-FU *in vivo*. **(A)** Immunohistochemistry was performed to detect the expression of Keap1 (magnification, × 400, scale bar, 20 μm). **(B)** A semiquantitative analysis of the ratio of Keap1-positive staining to the total field. **(C)** The nuclear translocation of Nrf2 was detected by immunofluorescence (IF) method, with Nrf2 antibody (red) and DAPI staining of the nucleus (blue) (magnification, × 400, scale bar, 20 μm). **(D)** Quantitative results of the percentage of Nrf2-positive nuclei in the livers. **(E)** The immunofluorescence results showed that ASP alleviated the inhibition of HO-1 activity caused by 5-FU in the livers of mice (magnification, × 400, scale bar, 20 μm). **(F)** The mean fluorescence intensity of HO-1 was quantified (*n* = 6/group). **(G–J)** Western blot assay was used to detect the cytoplasmic and nuclear levels of Nrf2 and the protein expression levels of Keap1 and HO-1in mouse livers (*n* = 3/group). The intensity of the bands was calculated using ImageJ software. β-actin and Lamin B1 (nuclear fraction) served as the internal control. ^*^p < 0.05; ^**^p < 0.01; ^***^p <0.001. n.s., not statistically significant.

The immunohistochemistry results showed that ASP treatment could relatively inhibit the increase in Keap1 expression induced by 5-FU (**Figures 5A, B**), which was further confirmed by western blot experiments (**Figures 5I, J**). Immunofluorescence results showed that 5-FU treatment hindered the nuclear translocation of Nrf2, and ASP treatment significantly enhanced the transcriptional activity of Nrf2; these results were further confirmed by western blot experiments (**Figures 5C, D, G, H**). The immunofluorescence results also showed that 5-FU treatment inhibited the relative expression of HO-1, and ASP treatment significantly promoted the expression of HO-1; these results were further confirmed by western blot experiments (**Figures 5E, F, I, J**). Overall, these results indicated that 5-FU enhanced oxidative stress by inhibiting the activity of Nrf2 and that ASP rescued the inhibitory effect of 5-FU on the Nrf2 pathway *in vivo*.

### ASP Treatment Reduced 5-FU-Induced Cell Oxidative Damage, the Inhibition of Lipid Metabolism Gene Expression and Cell Apoptosis *In Vitro*

**Figure 6:**
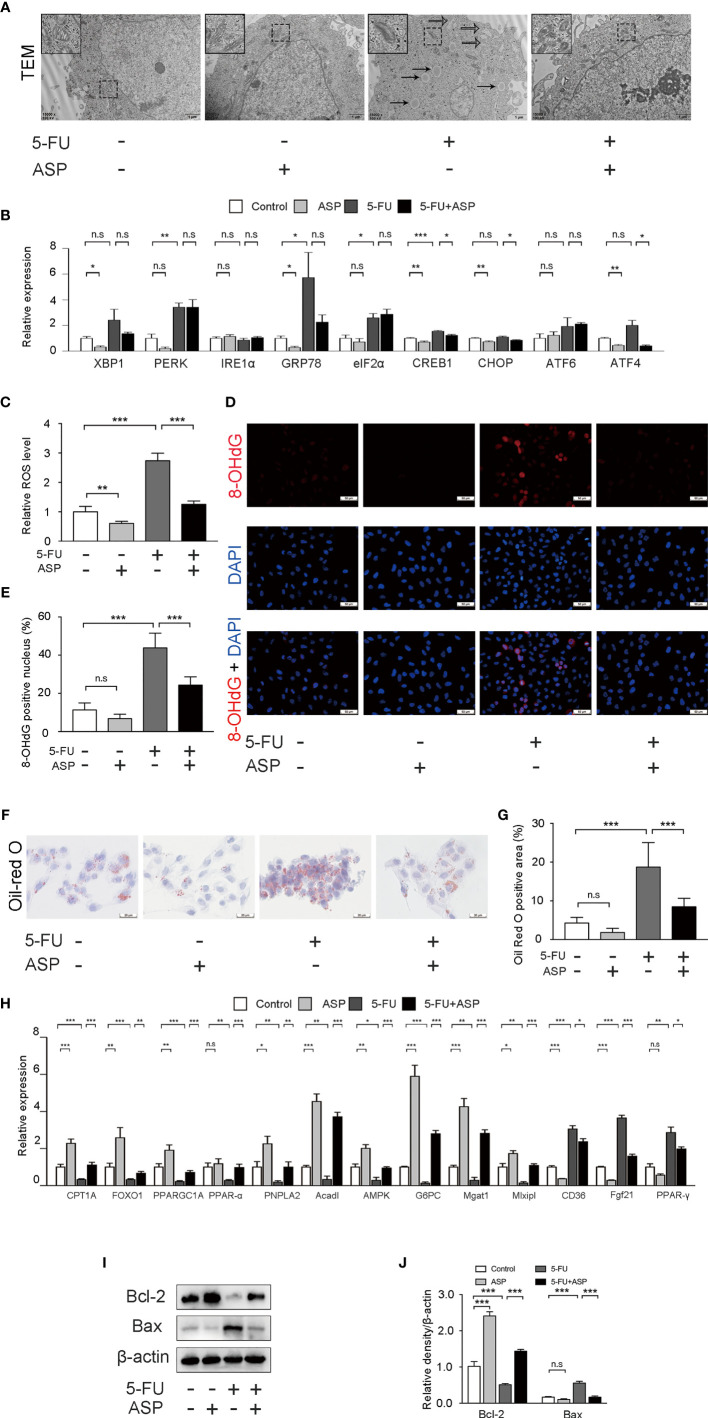
ASP treatment reduced 5-FU-induced cell oxidative damage and apoptosis and inhibited lipid metabolism gene expression *in vitro*. **(A)** Cell ultrastructure was observed under a TEM (magnification, × 15000, scale bar, 1 μm). The solid arrow points to the lipid droplet, and the open arrow points to the swollen endoplasmic reticulum. The swollen mitochondria are shown within the box at a higher magnification. **(B)** The expression of 9 genes related to endoplasmic reticulum stress was detected by RT-qPCR. β-actin was probed as an internal control (*n* = 3). **(C)** ROS production of the indicated groups (*n* = 6/group). **(D, E)** Representative immunofluorescence images and the quantification of 8-OHdG staining in cells (magnification, × 400, scale bar, 20 μm). **(F)** Oil Red O staining of cells (magnification, × 400, scale bar, 20 μm). **(G)** Oil Red O-positive areas were analyzed using ImageJ (*n* = 6/group). **(H)** The expression of lipid synthesis and metabolism-related genes in cells was detected by RT-qPCR assay (*n* = 3). β-actin was probed as an internal control. **(I)** Western blot assay was performed to determine the expression levels of Bax and Bcl-2. **(J)** The intensity of the bands was calculated using ImageJ software (*n* = 3/group). ^*^p < 0.05; ^**^p < 0.01; ^***^p < 0.001. n.s., not statistically significant.

Mitochondria are the primary source of ROS, and excessive production of ROS can lead to cell damage. The smooth endoplasmic reticulum is related to lipid metabolism. The fatty acids taken up by cells are decomposed by oxidoreductases in the smooth endoplasmic reticulum. Transmission electron microscopy results showed that a large amount of mitochondrial swelling and deformation was observed in the cytoplasm of the 5-FU (60 μg/mL) treatment group, the expansion of smooth endoplasmic reticulum was also observed, and there was a large area of lipid deposition. ASP (100 μg/mL) treatment reversed the effect of 5-FU on mitochondria, smooth endoplasmic reticulum and lipid deposition to a certain extent (**Figure 6A**). The expression of 9 genes related to endoplasmic reticulum stress was detected by RT-qPCR (**Figure 6B**). Compared with the control group, the expression of 8 genes in the 5-FU treatment group increased, and the expression of 1 gene decreased. The mRNA levels of XBP1, IRE1α, CHOP, ATF6, and ATF4 were not significantly different between the two groups. The expression of PERK, GRP78, eIF2α and CREB1 was increased significantly. RT-qPCR results demonstrated that ASP treatment could restore the increase in the expression of the 5-FU-induced endoplasmic reticulum stress-related genes CREB1, CHOP and ATF4 (**Figure 6B**). The ROS content in the 5-FU group was increased as compared with the control group, and ASP treatment partially eliminated intracellular ROS (**Figure 6C**). 8-OHdG is one of the major products of DNA oxidation. ASP treatment alleviated the 5-FU-induced increase in 8-OHdG levels in MIHA cells (**Figures 6D, E**). In MIHA cells, the Oil Red O-positive area increased significantly after 5-FU treatment for 24 h, and intracellular lipid droplets increased. After treatment with ASP, the changes were alleviated (**Figures 6F, G**), suggesting that ASP has an improvement effect on 5-FU-induced lipid deposition in MIHA cells. RT-qPCR results demonstrated that ASP treatment could significantly restore the decrease in the expression of the 5-FU-induced lipid synthesis and metabolism-related genes CPT1A, FOXO1, PPARGC1A, PPAR-alpha, PNPLA2, Acadl, AMPK, G6PC, Mgat1 and Mlxipl. And ASP treatment could significantly inhibit the increase in the expression of the 5-FU-induced CD36, Fgf21 and PPAR-γ (**Figure 6H**). In addition, the results indicated that the pro-apoptotic protein Bax was increased in the 5-FU group. In contrast, the anti-apoptotic protein Bcl-2 was significantly reduced; this situation was reversed after ASP treatment (**Figures 6I, J**), which indicated that 5-FU was inhibited by ASP treatment-induced hepatocyte apoptosis, which was consistent with the *in vivo* data. The above results suggest that ASP treatment can reduce 5-FU-induced cell oxidative damage and inhibit lipid metabolism gene expression and cell apoptosis *in vitro*.

### ASP Rescued the Inhibitory Effect of 5-FU on Nrf2 Pathway *In Vitro*

**Figure 7:**
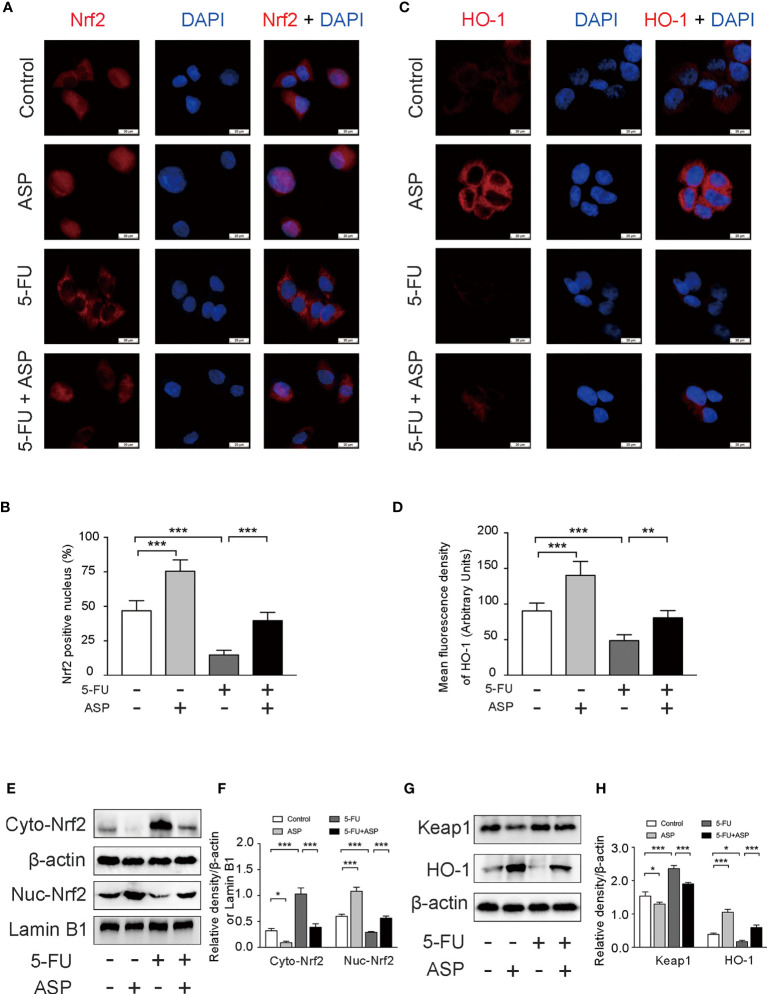
ASP rescued the inhibition of the Keap1- Nrf2 pathway caused by 5-FU *in vitro*. **(A)** The nuclear translocation of Nrf2 was detected by immunofluorescence (IF) method, with Nrf2 antibody (red) and DAPI staining of the nucleus (blue) (magnification, × 400, scale bar, 20 μm). **(B)** Quantitative results of the percentage of Nrf2-positive nuclei in the cells. **(C)** The immunofluorescence results showed that ASP alleviated the inhibition of HO-1 activity caused by 5-FU in cells (magnification, × 400, scale bar, 20 μm). **(D)** The mean fluorescence intensity of HO-1 was quantified (*n* = 6/group). **(E–H)** Western blot assay was used to detect the cytoplasmic and nuclear levels of Nrf2 and the protein expression levels of Keap1 and HO-1 in the cells (*n* = 3/group). The intensity of the bands was calculated using ImageJ software. β-actin and Lamin B1 (nuclear fraction) served as the internal control. ^*^p < 0.05; ^**^p < 0.01; ^***^p < 0.001.

The immunofluorescence results in MIHA cells showed that 5-FU treatment hindered the nuclear translocation of Nrf2, and ASP treatment significantly enhanced the transcriptional activity of Nrf2; these were further confirmed by western blot experiments (**Figures 7A, B, E, F**). The immunofluorescence results also showed that 5-FU treatment reduced the expression of HO-1, whereas ASP treatment enhanced the expression of HO-1; these results were further confirmed by western blot experiments (**Figures 7C, D, G, H**). The results demonstrated that ASP treatment reduced the increase in Keap1 expression induced by 5-FU (**Figures 7G, H**). Overall, these results indicated that 5-FU enhanced oxidative stress by inhibiting Nrf2 activity, and ASP rescued the inhibitory effect of 5-FU on the Nrf2 pathway *in vitro*.

## Discussion

Chemotherapy, as one of the most commonly used measures for treating malignant tumors, is widely used in the clinical treatment of tumors. Tumor therapeutic doses of chemotherapy drugs kill tumor cells while also damaging normal cells, resulting in damage to normal cells and organs (30). It has been reported that the chemotherapy drugs oxaliplatin, asparaginase, carmustine, mercaptopurine, and methotrexate can be hepatotoxicity. The mechanism of hepatotoxicity may be that chemotherapy drugs induce inflammation, liver cell apoptosis, oxidative stress damage, and abnormal liver metabolism (31–34). The chemotherapy drug 5-fluorouracil is widely used clinically to treat various tumors, including colorectal cancer, breast cancer, and liver cancer. However, liver toxicity caused by chemotherapy often prevents the administration of sufficient effective drug doses to patients (4). Studies have shown that 5-FU treatment induces portal fibrosis and increased apoptosis, and at the ultrastructural level, vesicular rough endoplasmic reticulum and atrophic mitochondria are observed (7). Our previous studies have proven that the chemotherapy drug 5-FU can cause oxidative damage to bone marrow stromal cells and changes in the secretion of biologically active substances. The mechanism of 5-FU injury to the liver is still unclear and remains to be further explored and studied (2). Our results proved that ASP could reduce 5-FU-induced liver oxidative damage and watery degeneration, lipid accumulation, and vacuolation. As a result, the mitochondria of liver cells and the endoplasmic reticulum swell, thereby delaying cell apoptosis and reducing hepatotoxicity. ASP treatment significantly promotes nuclear translocation of liver Nrf2 protein, reduces liver oxidative stress levels, and reverses 5-FU-mediated liver pathological changes *in vivo* and *in vitro*.

Damages such as oxidants, γ-ray radiation and chemotherapy can put cells in oxidative stress, leading to abnormal metabolism and the apoptosis of cells (35, 36). In this study, 5-FU induced a loss of liver weight and increased TG, AST, and ALT levels, suggesting decreased liver function and triglyceride deposition in mice. Through various histomorphological staining techniques, severe lipid deposition and a large number of hepatocytes, swelling, necrosis, vacuolation and fibrosis were observed in the livers of the 5-FU group. Our study showed many fatty acid deposits and abnormal mitochondria in the 5-FU group *in vivo* and *in vitro*. In this experiment, 5-FU treatment caused damage to normal liver cells. However, the mechanism is not precise.

Many experiments have shown that oxidative stress can lead to endoplasmic reticulum stress, which induces apoptosis (37–39). Endoplasmic reticulum is an essential place for lipid synthesis. Abnormal endoplasmic reticulum function can cause disorders of lipid anabolism (40–42). In this study, by observing the ultrastructure of liver cells, we observed that after 5-FU treatment, the endoplasmic reticulum structure in the liver cells was abnormal, mainly manifested as swelling. RT-qPCR was used to detect the expression of 9 endoplasmic reticulum stress-related genes, including XBP1, PERK, IRE1α, GRP78, eIF2α, CREB1, CHOP, ATF6, and ATF4. The expression of 8 genes in the 5-FU treatment group increased, and the expression of 1 gene decreased compared with the control group. The mRNA levels of XBP1, IRE1α, CHOP, ATF6, and ATF4 were not significantly different between the two groups. The expression of PERK, GRP78, eIF2α, and CREB1 was increased significantly. RT-qPCR results also demonstrated that ASP treatment could restore the increase in the expression of the 5-FU-induced endoplasmic reticulum stress-related genes CREB1, CHOP, and ATF4. These results suggest that ASP can alleviate the endoplasmic reticulum stress caused by 5-FU. In addition, RT-qPCR was measured to detect the expression of 18 lipid synthesis-, uptake- and metabolism-related genes in mouse livers. The expression changes of 13 genes related to synthesis and metabolism in the 5-FU group were significantly different from the control group. This suggests that endoplasmic reticulum stress can indeed cause disorders of lipid synthesis and metabolism. Among them, PPAR-alpha can metabolize NEFAs that the liver has taken up, thereby protecting the liver from lipid peroxidation damage (43). PPAR-alpha is a nuclear receptor activated by a ligand. PPAR-alpha is the main lipid oxidation factor in the liver. PPAR-alpha regulates target genes involved in β oxidation, such as Cpt1a. PPAR-alpha promotes lipid oxidation in liver cells, thereby reducing lipids in animal models. Metabolism and steatosis are beneficial (44, 45). Ppargc1a is a crucial physiological transcription regulator of oxidative metabolism and mitochondrial biogenesis (46). Ppargc1a promotes mitochondrial biogenesis, oxidation capacity and fatty acid β oxidation by increasing the expression and activation of various transcription factors (such as PPAR-alpha) (47). In terms of fatty acid decomposition, the key TG hydrolase in the liver is Pnpla2, and Sirt1 can regulate fat mobilization through Foxo1-mediated Pnpla2 expression (48–50). Therefore, we focused on verifying the expression of five lipid-metabolism-related genes *in vitro*, and the results obtained were consistent with those of the *in vivo* experiments. After 5-FU treatment, the expression of 5 genes related to lipid metabolism decreased significantly. It has been suggested that 5-FU can induce oxidative stress to cause abnormal endoplasmic reticulum function to affect lipid synthesis, inhibit the expression of lipid metabolism genes, and ultimately lead to lipid deposition. Studies have also demonstrated that lipid deposition aggravates the production of ROS, which leads to cell apoptosis (51–53). In our experiment, we also observed that after 5-FU treatment, the apoptotic cells in the mouse liver increased. Western blot results further demonstrated the upregulation of Bax and the downregulation of Bcl-2 in the 5-FU group both *in vivo* and *in vitro*. However, there are few drugs target antiapoptotic proteins for clinical treatment. The occurrence of apoptosis is closely related to oxidative stress. Theoretically, eliminating oxidative stress can help alleviate apoptosis. Therefore, finding an effective, low-toxicity natural medicine that can exert antioxidant effects is necessary.

Under normal circumstances, the ROS content remains in balance (54, 55). Excessive ROS content will cause oxidative stress, leading to inflammation, apoptosis, and other disease processes (56, 57). Additionally, free fatty acids can cause lipotoxicity by disrupting the balance of the reactive oxygen system (58). Excessive production of ROS can cause lipid peroxidation and a disturbance of antioxidant and peroxidase activity to cause cell dysfunction and cell apoptosis (59, 60). Since the mitochondria can generate most of the ROS, mitochondria are considered the main contributors to oxidative stress (61, 62). In this study, 5-FU induced an increase in ROS levels both *in vivo* and *in vitro*. Furthermore, the ultrastructure of the liver and hepatocytes was observed. The liver mitochondria treated with 5-FU were swollen, and most of the mitochondria in the liver cells were abnormal. We speculate that 5-FU can cause a large amount of ROS production by damaging mitochondria morphology and causing dysfunction. The enzyme activities of SOD, GSH and CAT are essential indicators to assess the capacity to resist oxidation. In this study, 5-FU-induced a decrease in the enzyme activity of SOD, GSH, and CAT, suggesting that 5-FU caused damage to the liver’s antioxidant capacity. MDA is an end product of lipid peroxidation, a good marker of oxidative damage. NO is a highly reactive free radical in biology. In this study, 5-FU-induced an increase in the levels of MDA and NO, suggesting that 5-FU aggravates oxidative stress in the liver. In addition, the level of ROS in cells rises uncontrollably, which can induce the accumulation of DNA damage to promote the apoptosis process (63–65). Oxidative stress triggers DNA damage, leading to the DNA damage response (DDR). 8-OHdG is one of the major products of DNA oxidation (66, 67). Our results indicated that after 5-FU treatment of hepatocytes, the expression of 8-OHdG was significantly increased, suggesting that 5-FU-induced oxidative stress caused DDR. Our results show that 5-FU can damage the mitochondrial function and increase ROS production, which in turn reduces the activity of antioxidant enzymes and increases peroxides content, leading to DNA damage and DDR, thereby promoting cell apoptosis.

Nrf2 is a crucial regulator in the cellular oxidative stress response (68), regulated by Keap1 to regulate the expression of antioxidant proteins (69). It has been found that the Nrf2 signaling pathway is one of the core pathways of the cellular antioxidant response, which can significantly induce the body’s endogenous antioxidant response (70). The abnormal Nrf2 signaling pathway will aggravate oxidative stress damage and destroy the normal redox balance in the cell (71, 72). Keap1 protein is sensitive to oxidative stress. Under normal conditions, Keap1 and Nrf2 exist as dimers in the cytoplasm. Once exposed to oxidative stress, the two dissociate and Nrf2 is transcribed to regulate the transcriptional activation of a series of cytoprotective genes (73), such as heme oxygenase-1 (HO-1) (74). HO-1 has anti-apoptosis, anti-inflammatory, and antioxidant functions (75, 76). The effect of 5-FU on the Nrf2 pathway and its mechanism remains to be explored. We detected the expression of Nrf2 pathway proteins in mouse livers and MIHA cells. The results showed that in both mice and cells treated with 5-FU, the level of Keap1 was relatively increased. In contrast, the level of HO-1 was decreased, which was further confirmed by immunohistochemistry experiments and immunofluorescence experiments. The results of immunofluorescence and western blot experiments showed that after 5-FU induction, the level of Nrf2 in the nucleus was significantly reduced, and the transport of Nrf2 into the nucleus was reduced. It is suggested that 5-FU induction can inhibit Nrf2 nuclear translocation, thereby reducing the level of the downstream antioxidant protein HO-1, leading to further weakening of the antioxidant ability of cells and intensifying oxidative stress.

ASP is extracted from the root of Dang Gui. The main components of ASP include galactonic acid, glucuronic acid, galactose, arabinose, xylose, and rhamnose (77, 78). It has been proven that ASP has beneficial effects in many disease models, such as diabetes, osteoarthritis, and anemia (79–81). Our previous studies have shown that ASP slows down the senescence of bone marrow stromal cells and hematopoietic cells by inhibiting oxidative stress (2, 25). A study showed that angelica polysaccharides could reduce CCl4-induced liver fibrosis (82). However, to date, the effect of ASP on 5-FU-induced liver hepatotoxicity and the mechanism by which it occurs is still unknown.

This study demonstrated that ASP protects the liver from structural and functional damage caused by 5-FU, mainly by reducing the symptoms of apoptosis. ASP reversed the nuclear translocation disorder of Nrf2 caused by 5-FU, leading to increased expression of antioxidant genes to inhibit oxidative stress, restoring the function of the mitochondria and endoplasmic reticulum to reduce lipid deposition and DNA damage, and alleviating apoptosis. This study provides not only a protective agent for liver injury caused by the chemotherapy drug 5-FU but also a novel target for treating the liver injury.

## Data Availability Statement

The original contributions presented in the study are included in the article/**Supplementary Material**. Further inquiries can be directed to the corresponding author.

## Ethics Statement

The animal study was reviewed and approved by Chongqing Medical University Animal Care and Use Committee.

## Author Contributions

DZ, YW, LW, and JL designed the study. DZ, HX, ZW, GL, and YC performed the experiments. HL, JH, and DL analyzed the results. DZ, YW, and JL wrote the manuscript. All authors contributed to the article and approved the submitted version.

## Funding

This study was supported by the National Natural Science Foundation of China (Nos. 81873103, 81673748).

## Conflict of Interest

The authors declare that the research was conducted in the absence of any commercial or financial relationships that could be construed as a potential conflict of interest.

## Publisher’s Note

All claims expressed in this article are solely those of the authors and do not necessarily represent those of their affiliated organizations, or those of the publisher, the editors and the reviewers. Any product that may be evaluated in this article, or claim that may be made by its manufacturer, is not guaranteed or endorsed by the publisher.
