# Supplementary material for: Angelica Polysaccharide Antagonizes 5-FU-Induced Oxidative Stress Injury to Reduce Apoptosis in the Liver Through Nrf2 Pathway
Source: Front Oncol. 2021 Aug 16;11:720620. doi: 10.3389/fonc.2021.720620 (PMC8415481; doi:10.3389/fonc.2021.720620)
Supplement: Supplementary file 1 [file Table_1.doc]

|  | **Table 1**. Primer sequences for RT-qPCR | | |
| --- | --- | --- | --- |
| Species | Gene | Sequence | 5' to 3' |
| Mouse | Mlxipl | Forward | TACCTCCTTCGTGCTCTC |
|  |  | Reverse | AGGCTGGTCTCACTCATTA |
| Mouse | PPAR-γ | Forward | CCACCAACTTCGGAATCA |
|  |  | Reverse | GCTCTTGTGAATGGAATGTC |
| Mouse | Scd1 | Forward | CTGTTCGTTAGCACCTTCT |
|  |  | Reverse | GGATTGAATGTTCTTGTCGTAG |
| Mouse | G6pc | Forward | AAGGATGGAGGAAGGAAT |
|  |  | Reverse | CACAGACACAAGGATGAA |
| Mouse | Mgat1 | Forward | AAGGTATTGCCATTCTAATCCA |
|  |  | Reverse | TGCCTCAGTTCCTCTTCT |
| Mouse | Dgat1 | Forward | AGATTGGTGGAATGCTGAG |
|  |  | Reverse | ATGGAAGAAGGCTGAGGT |
| Mouse | Dgat2 | Forward | GGTGCCTTCTGTAACTTCA |
|  |  | Reverse | TCTATGGTGTCTCGGTTGA |
| Mouse | PPAR-alpha | Forward | ACATAGGCTACATTGAGAAGTT |
|  |  | Reverse | AGGAAGGTGTCATCTGGAT |
| Mouse | Cpt1a | Forward | CAAGCCAGACGAAGAACA |
|  |  | Reverse | TGACCATAGCCATCCAGAT |
| Mouse | Pnpla2 | Forward | TTCAGACAACTTGCCACTT |
|  |  | Reverse | CGGTAGAGATTGCGAAGG |
| Mouse | Acadl | Forward | CTCAGGACACAGCAGAAC |
|  |  | Reverse | AATCGCCAACTCAGCAAT |
| Mouse | Ppargc1a | Forward | ACAACAATAACAACAACAACAAC |
|  |  | Reverse | GTGCTTACTGGATTATCATTCTG |
| Mouse | Fgf21 | Forward | CCAAGACCAAGCAGGATT |
|  |  | Reverse | GAAGAGTCAGGACGCATAG |
| Mouse | AMPK | Forward | TTCCGTAGTATTGATGATGAGAT |
|  |  | Reverse | GAGGTGACAGATGAGGTAAG |
| Mouse | Foxo1 | Forward | GCTCTGTCCTGAAGAATCC |
|  |  | Reverse | ATCCTGCCACTGTCTGTA |
| Mouse | Sirt1 | Forward | CCACCAACACCTCTTCATAT |
|  |  | Reverse | ACACAACTTGATTCAGATACTTC |
| Mouse | Ldlr | Forward | CTACTGGCTGTGCTTGAA |
|  |  | Reverse | CCTCCGTGTTAGTGTTGAA |
| Mouse | Cd36 | Forward | AAGTTGCCATAATTGAGTCCTA |
|  |  | Reverse | CCGAACACAGCGTAGATAG |
| Mouse | β-ACTIN | Forward | AGATTACTGCTCTGGCTCCTAGC |
|  |  | Reverse | ACTCATCGTACTCCTGCTTGCT |
| Human | CPT1A | Forward | GATTTCCATTCCTTCCCATTCG |
|  |  | Reverse | CTCGTATGTGAGGCAAAACTTG |
| Human | FOXO1 | Forward | AAACACCAGTTTGAATTCACCC |
|  |  | Reverse | TCGACTTATTGTCCTGAAGTGT |
| Human | PPARGC1A | Forward | CAGAGAGTATGAGAAGCGAGAG |
|  |  | Reverse | AGCATCACAGGTATAACGGTAG |
| Human | PPAR-α | Forward | AACTTCAACATGAACAAGGTCAAAGCC |
|  |  | Reverse | CAGCGTCTTCTCAGCCATACACAG |
| Human | PNPLA2 | Forward | CTGCCACTCTATGAGCTTAAGA |
|  |  | Reverse | TATCCCTGCTTGCACATCTC |
| Human | β-ACTIN | Forward | AGAAAATCTGGCACCACACCT |
|  |  | Reverse | GATAGCACAGCCTGGATAGCA |
| Human | GRP78 | Forward | CCTACCAAGAAGTCTCAG |
|  |  | Reverse | ATACCATTCACATCTATCTCA |
| Human | IRE1α | Forward | TGGAAGCAAGAATAATGAAG |
|  |  | Reverse | GTCAGGAGGTCAATAACA |
| Human | ATF6 | Forward | AGTTATTCAGTCTCGTCTC |
|  |  | Reverse | GGCTTATCTTCCTTCAGT |
| Human | PERK | Forward | AATGAGAACACAGAAGAGT |
|  |  | Reverse | TAAGTAACCAGGCAGATG |
| Human | CREB1 | Forward | AGTCAGTGGATAGTGTAAC |
|  |  | Reverse | GCATCAGAAGATAAGTCATT |
| Human | eIF2α | Forward | AAGATGTAGTGATGGTGAA |
|  |  | Reverse | GATACGCCTTCTGGATAA |
| Human | ATF4 | Forward | ACCTTCTTACAACCTCTTC |
|  |  | Reverse | GCTTCCTATCTCCTTCAG |
| Human | CHOP | Forward | TTAAGTCTAAGGCACTGAG |
|  |  | Reverse | TGTGGTGATGTATGAAGATA |
| Human | XBP1 | Forward | AACAGCAAGTGGTAGATT |
|  |  | Reverse | CTTAACTCCTGGTTCTCAA |
| Human | Acadl | Forward | AAGTGATGTTGTGATTGTAG |
|  |  | Reverse | AATAGTTCTGCGGTATCC |
| Human | AMPK | Forward | TCCGTAGTATTGATGATGAA |
|  |  | Reverse | CACAGATGAGGTAAGAGAA |
| Human | G6PC | Forward | AAGAAGTCGTTGTAAGAGA |
|  |  | Reverse | AATAGTAGTCCTCCTCAATC |
| Human | Mgat1 | Forward | CTCGGATACTGTGTTCTGT |
|  |  | Reverse | CCTTGGATGCTGATGAGT |
| Human | Mlxipl | Forward | GGTATATCCAGTATGTGAAG |
|  |  | Reverse | CGCTTCTTGTAGTAGATG |
| Human | CD36 | Forward | GTTGGAGACCTGCTTATC |
|  |  | Reverse | CTGCTGTTCATCATCACT |
| Human | Fgf21 | Forward | GGAACCTCAGTGTCAGAT |
|  |  | Reverse | ATCACGCAGAACAAGAGA |
| Human | PPAR-γ | Forward  Reverse | GCATCTCCACCTTATTATTC  ACAGACACGACATTCAAT |
|  |  |  |  |
